# Changes of intestinal bacterial microbiota in coronary heart disease complicated with nonalcoholic fatty liver disease

**DOI:** 10.1186/s12864-019-6251-7

**Published:** 2019-11-14

**Authors:** Yiwen Zhang, Jun Xu, Xuemei Wang, Xinhua Ren, Yulan Liu

**Affiliations:** 1 Department of Gastroenterology, Peking University People’s Hospital, No.11 Xizhimen South Street, Xicheng District Beijing, People’s Republic of China; 2 Clinical Center of Immune-Mediated Digestive Diseases, Peking University People’s Hospital, No.11 Xizhimen South Street, Xicheng, Beijing, People’s Republic of China; 3 Institute of Clinical Molecular Biology & Central Laboratory, Peking University People’s Hospital, No.11 Xizhimen South Street, Xicheng District, Beijing, People’s Republic of China

**Keywords:** Non-alcoholic fatty liver disease, Coronary heart disease, Intestinal microbiota

## Abstract

**Background:**

Previous study reported that patients who suffered coronary heart disease (CHD) complicated with non-alcoholic fatty liver disease (NAFLD) had worse cardiac function and clinical outcomes than patients with CHD only. Notably, the mechanism is still unclear. This study aimed to investigate the changes and roles of intestinal bacterial microbiota in CHD-NAFLD patients.

**Methods and results:**

People were recruited and divided into three groups, including CHD patients (without NAFLD), CHD-NAFLD patients and healthy controls (HCs). Each group contained 24 people. Fecal samples and clinical information were carefully collected. The Illumina sequencing of 16S rRNA was applied to profile the overall structure of the fecal bacterial microbiota and the characteristics of the bacterial microbiota based on the Operational Taxonomic Units. In clinical information, the CHD-NAFLD patients showed an increase in BMI, uric acid and triglyceride. There was a significant reduction in the abundance of *Parabacteroides* and *Collinsella* in overall CHD patients (including CHD-NAFLD and CHD patients). The intestinal bacterial microbiota in CHD-NAFLD patients showed an increase in the abundance of *Copococcus* and *Veillonella*, and a reduction in the abundance of *Parabacteroides*, *Bacteroides fragilis*, *Ruminococcus gnavus, Bacteroides dorei*, and *Bifidobacterium longum subsp infantis*. Among them, the abundance of *Ruminococcus gnavus* and *Bacteroides dorei* was significantly lower than that in CHD patients. Additionally, BMI positively correlated with the abundance of *Copococcus* and negatively correlated with the abundance of *Bifidobacterium longum subsp infantis*. The abundance of *Veillonella* positively correlated with AST. The abundance of *Bacteroides dorei* negatively correlated with ALT and AST. It indicates that the abundance of intestinal microbiota was related to the changes in clinical indexes.

**Conclusions:**

Changes of intestinal bacterial microbiota in CHD-NAFLD patients may be important factors affecting the degree of metabolic disorder, which may be one of the important reasons for the worse clinical outcome and disease progression in CHD-NAFLD patients than in CHD patients.

## Background

It was reported that nonalcoholic fatty liver disease (NAFLD) had a certain correlation with coronary atherosclerotic heart disease (CHD). A number of epidemiological studies found that NAFLD might increase the risk of cardiovascular disease [1]. A prospective observational study including 1637 Japanese subjects found that the incidence of atherosclerotic cardiovascular disease including CHD and ischemic stroke was significantly higher in CHD patients complicated with NALFD (CHD-NAFLD) than in CHD patients [2]. Moreover, it was found that the incidence and mortality of cardiovascular events in NAFLD patients significantly increased [3, 4]. For CHD-NAFLD patients, the rate of coronary stenosis was higher than that in CHD patients without NAFLD [5] and the severity of CHD and cardiac function were worse than those in CHD patients [6]. There are few studies on the mechanisms, especially from the perspective of intestinal microbiota.

CHD is currently the leading cause of death in western countries. Metabolic diseases, as risk factors for coronary heart disease, such as diabetes, obesity and NAFLD, were also increasing in prevalence worldwide. A large number of recent studies have focused on the role of intestinal microbiota in CHD [7] and there was also continuous evidence that intestinal microbiota was closely related to atherosclerosis. The drug for CHD targeted on the intestinal microbiota had also made some progress [8].

NAFLD gradually becomes one of the most common chronic liver diseases worldwide [9, 10]. At present, the pathogenesis of NAFLD is still unclear. In recent years, it was believed that NAFLD tended to be caused by various factors including genetic differences, insulin resistance, intestinal microbial dysbiosis and lipid metabolism [11]. At present, it has been found that the intestinal microbiota also played a certain role in the occurrence and development of NAFLD [12].

Gut microbiota and metabolism play pivotal roles in the progression of CHD and NAFLD. It was speculated that the intestinal microbiota played an important role in the progression and outcome of CHD-NAFLD patients. The intestinal microbiota in CHD-NAFLD patients might be different from that in CHD patients. This study was designed to investigate the characteristics and effects of intestinal microbiota in CHD-NAFLD patients.

## Results

### Clinical characteristics

We have included three groups of 72 patients, 24 in each group. The ratio of male to female is 17/7 and the age and gender of the three groups of patients were matched. To be mentioned, though 72 patients were recruited, the microbiota information of one person in the 24 HCs was missed. So in the analysis of microbiota, 71 samples were used. The basic information is shown in (Table 1).

**Table 1:** Clinical characteristics of the patients

	CHD-NAFLD (*N* = 24)	CHD (*N* = 24)	HC (*N* = 23)
Male/Female (N)	17/7	17/7	16/7
Age(Mean ± SD)	63.54 ± 7.21	63.50 ± 7.70	64.04 ± 7.30
BMI	27.74 ± 2.72^*#^	24.46 ± 5.80	24.83 ± 4.32
HBP (N)	18	17	10
DM (N)	11	6	9
Smoke (N)	15	14	5
ALT	25.04 ± 11.69	20.45 ± 13.28	18.09 ± 10.23
AST	25.54 ± 12.97	20.45 ± 12.73	21.13 ± 10.00
GGT	40.96 ± 34.11	31 ± 26.55	27.23 ± 33.85
ALP	80.17 ± 18.02	77.33 ± 20.30	82.52 ± 24.71
UA	405.21 ± 103.08^*^	371.33 ± 112.13	331.04 ± 76.64
BUN	5.50 ± 1.56	5.89 ± 2.00	5.34 ± 1.04
HDL-C	1.04 ± 0.34	1.03 ± 0.24	1.09 ± 0.26
LDL-C	2.45 ± 0.67	2.37 ± 0.72	2.55 ± 0.88
TG	1.88 ± 1.69^*^	1.40 ± 0.79	1.12 ± 0.52
Cre	72.58 ± 19.11	82.79 ± 31.71	70.26 ± 16.94
EF %	64.58 ± 7,11	66.35 ± 6.61	67.87 ± 5.04
NCA	1.78 ± 0.85	1.63 ± 1.10	
HMI	6	5	
Statin	24	24	11

*BMI* Body mass index, *HBP* High blood pressure, *DM* diabetes mellitus, *ALT* Alanine aminotransferase, *AST* Aspartate aminotransferase, *GGT* Glutamyl transpeptidase, *ALP* Alkaline phosphatase, *UA* uric acid, *BUN* Blood urea nitrogen, *HDL-C* High-density lipoprotein cholesterol, *LDL-C* Low-density lipoprotein cholesterol, *TG* Triglyceride, *Cre* creatinine, *EF* ejection fractions, *NCA* Narrowed coronary artery, *HMI* History of myocardial infarction

* *p* < 0.05, CHD-NAFLD patients vs HCs

# p < 0.05, CHD-NAFLD patients vs CHD patients

The levels of uric acid and triglyceride in CHD patients were higher than those in HCs. These clinical indexes in CHD-NAFLDD patients were further increased, which was significantly higher than HCs (*p* < 0.05). The BMI of CHD patients was not significantly different from that of the HCs, but the BMI of CHD-NAFLDD patients was significantly higher than that of the HCs (*p* < 0.0.5). These results indicated that the changes of BMI in CHD-NAFLDD patients are higher than those in CHD patients. Though the Uric acid and triglyceride in CHD-NAFLD was not significantly higher than that in CHD patients, we have observed the trends of increasing of the Uric acid and triglyceride in CHD-NAFLD patients compared with CHD patients.

In terms of cardiac function, the echocardiographic ejection fraction of CHD-NAFLD patients was lower than that of CHD patients. The number of narrowed coronary artery was higher than that of CHD patients. The narrowed coronary artery was defined as the coronary artery with more than 70% stenosis including left main coronary artery, left anterior descending artery, left circumflex artery and right coronary artery.

### Diversity of the fecal microbiota

We used Shannon index and chao1 index to assess the ɑ-diversity of the microbiota. Principal coordinate analysis (PCoA) was used for the β-diversity of the microbiota.

**Fig. 1:**
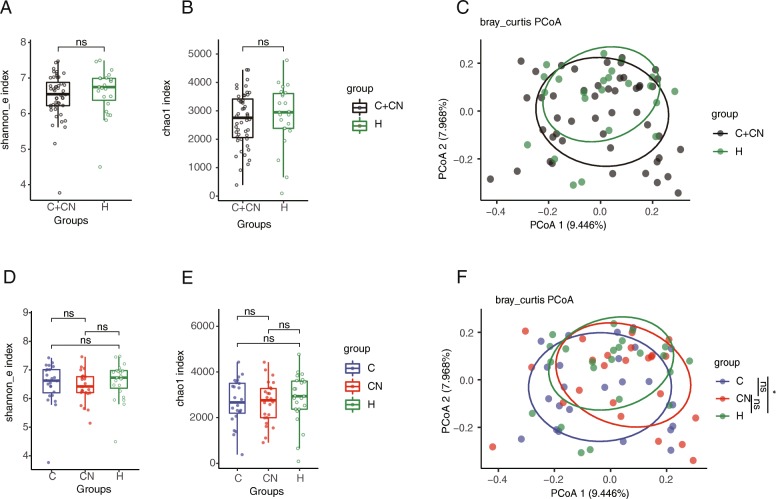
The diversity of the fecal microbiota. (**a**) The Shannon index in the overall CHD patients. (**b**) The Chao1 index in the overall CHD patients. (**c**) The β diversity of the overall CHD patients based on the PCoA analysis. (**d**) The Shannon index in CHD-NAFLD patients. (**e**) The Chao1 index in CHD-NAFLD patients. (**f**) The β diversity of the CHD-NAFLD patients based on the PCoA analysis. The “CN” stood for CHD-NAFLD patients. The “C” stood for CHD patients. The “H” stood for HCs. The “C + CN” stood for the overall CHD patients. *p < 0.05; .*p* < 0.1. 71 samples were used in each analysis. Kruskal-Wallis H test was used in the comparsion of Shannon index and Chao1 index. In the comparsion of PCoA analysis, adonis test was used

The differences of Shannon and chao1 indexes between the overall CHD patients and the HCs were analyzed and were not statistically different (Fig. 1a and b). The PCoA analysis showed that there was a certain difference in the composition pattern of the bacterial microbiota between the overall CHD patients and the HCs, although it was not statistically significant (*p* = 0.08) (Fig. 1c). The difference of Shannon index and chao1 index between CHD patients, CHD-NAFLD patients and the HCs was also not statistically different (Fig. 1d and e). For the β diversity, the difference in PCoA between the CHD patients and HCs was statistically significant. For the CHD-NAFLD patients, the PCoA analysis showed no significant difference with either CHD patients or HCs (Fig. 1f). These results didn’t show a distinctive bacterial composition in different groups.

### The microbiota at phylum and genus level

**Fig. 2:**
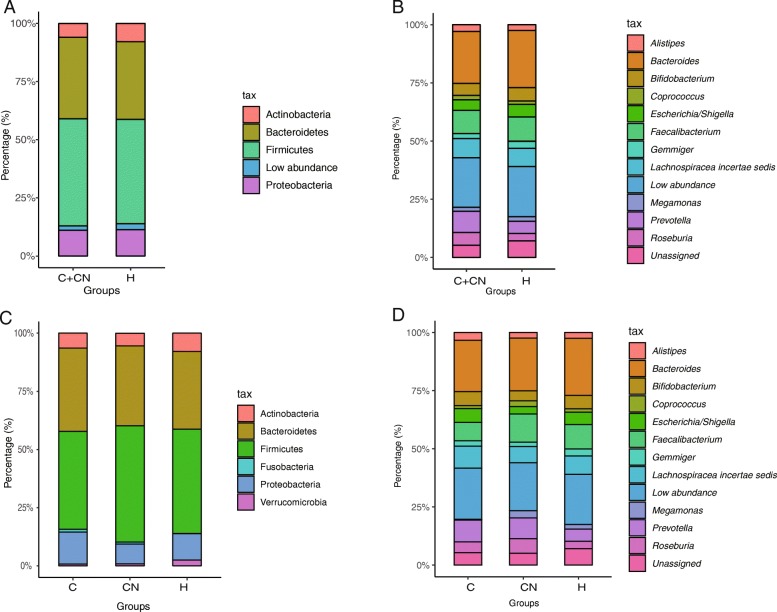
The composition of the bacteria at the phylum and genus level. (**a**) The bacteria at the phylum level in the overall CHD patients. (**b**) The bacteria at the genus level in the overall CHD patients. The top 13 genus in abundance was listed. (**c**) The bacteria at the phylum level in the CHD-NAFLD and CHD patients. (**d**) The bacteria at the genus level in the CHD-NAFLD and CHD patients. The low abundance stood for the abundance of other unlisted phylum and genus. The listed phylum and genus were the top 4 phylum and top 11 genus in abundance. 71 samples were used in each analysis

Among all the identified OTUs, the Firmicutes and Bacteroidetes phyla were the two most abundant phylum in the overall CHD patients and HCs (Fig. 2a). For the CHD patients and CHD-NAFLD patients, the Firmicutes and Bacteroidetes were also the dominant phylum (Fig. 2c).

At the genus level, the composition of bacterial microbiota of the overall CHD patients and HCs was analyzed (Fig. 2b). *Bacteroides*, *Faecalibacterium*, *Prevotella*, *Roseburia*, *Bifidobacterium*, *Escherichia/Shigella*, *Megamonas*, *Alistipes*, *Gemmiger* were the main genus of the bacterial microbiota in the overall CHD patients and HCs. Among them, *Bacteroides* (the percentage of *Bacteroides* in overall CHD patients and HCs: 22.3779 and 24.5413) and *Bifidobacterium* (the percentage of *Bifidobacterium* in overall CHD patients and HCs: 5.1405 and 5.7545) had a lower abundance in the overall CHD patients than in the HCs, though it was not statistically significant. Previous studies reported that *Bacteroides* and *Bifidobacterium* were mostly protective bacteria for metabolic diseases [13, 14].

For the CHD patients and CHD-NAFLD patients, *Bacteroides*, *Faecalibacterium*, and *Prevotella* were also the main genus of the bacterial microbiota (Fig. 2c). The abundance of *Coprococcus* increased in the CHD-NAFLD patients, though it was not statistically significant (the abundance of *Coprococcus* in CHD-NAFLD, CHD, HCs: 2.4892, 1.3141, 1.4745). Previous studies have reported that *Copulococcus* had a close relationship with metabolic syndrome and atherosclerosis [15]. These data indicated that the changes in the abundance of bacteria in either overall CHD patients or CHD-NAFLD patients might be related to the metabolism.

### The characteristic of bacterial microbiota of the overall CHD patients

**Fig. 3:**
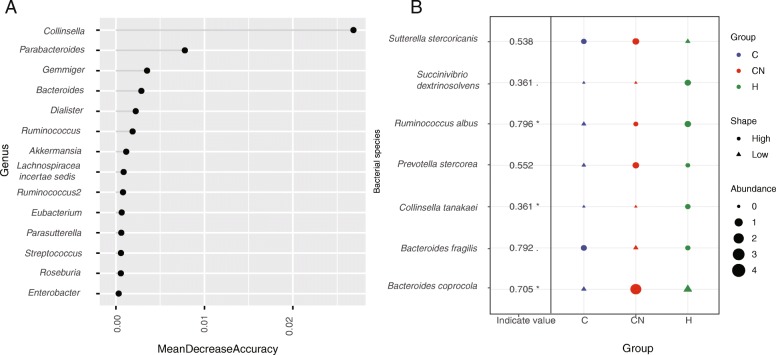
The specific microbiota at the genus and species level. (**a**) The specific bacterial microbiota at the genus level in the overall CHD patients. The random forest analysis was used. The MeanDecreaseAccuracy contained a measure of the extent to which a variable improves the accuracy of the forest in predicting the classification. Higher values mean that the variable improves prediction. (**b**) The specific bacterial microbiota at the species level. The R3.5.1 with *indicspecies* package was used. Permutation test was performed. The shape of the graph represents the comparison in enrichment (circle) or depletion (triangle) between three groups. The size of the graph indicates the relative abundance. *p < 0.05; .*p* < 0.1. 71 samples were used in each analysis

In order to improve the accuracy of the model, all patients were included in the model construction with no prediction. The random forest was used to analyze the specific bacteria at the genus level. We found that the abundance of *Collinsella* and *Parabacteroides* in the overall CHD patients was lower than in HCs, which was the characteristic bacteria of the overall CHD patients (Fig. 3a).

At the species level, indicating species found that the abundance of *Collinsella tanakaei* in the overall CHD patients was lower than that in HCs, which was the characteristic of the bacteria microbiota of the overall CHD patients (Fig. 3b). We also found the abundance of *Sutterella stercoricanis* was higher than that in HCs, though it was not statistically significant. Among them, previous studies suggested that *Parabacteroides* have a certain protective effect on metabolism [16]. The *Sutterella stercoricanis* was reported to be associated with obesity and liver damage [17]. It suggests that the changes in the abundance of *Parabacteroides* and *Collinsella* was the characteristics of the bacterial microbiota of the overall CHD patients.

### The characteristic of bacterial microbiota of the CHD patients

**Fig. 4:**
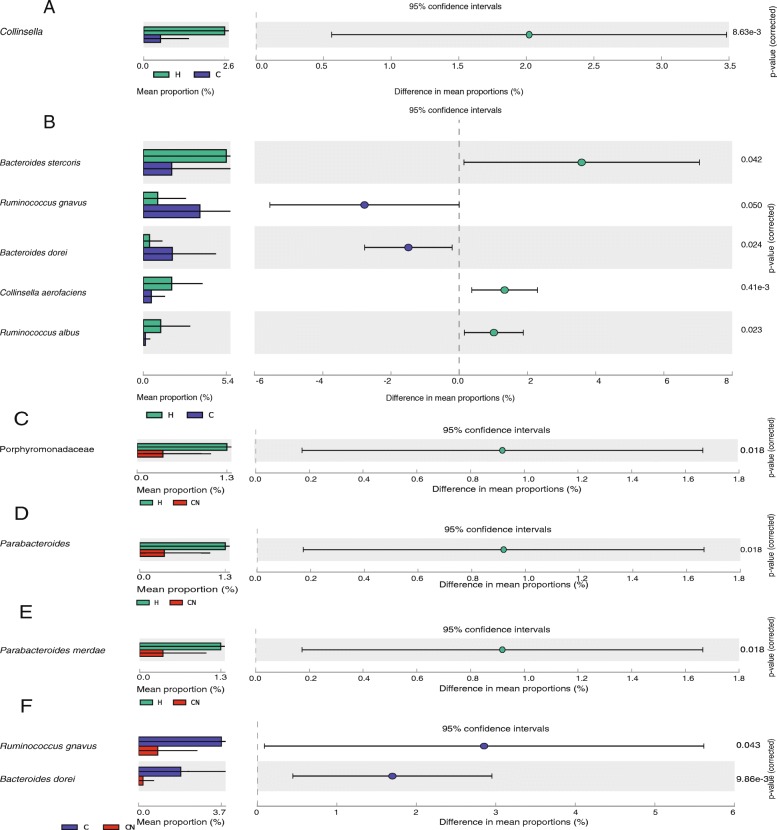
The comparison of bacterial microbiota in CHD and CHD-NAFLD patients. (**a**) The comparison of bacterial microbiota between CHD patients and HCs at genus level. (**b**) The comparison of bacterial microbiota between CHD patients and HCs at species level. (**c**) The comparison of bacterial microbiota between CHD-NAFLD patients and HCs at family level. (**d**) The comparison of bacterial microbiota between CHD-NAFLD patients and HCs at genus level. (**e**) The comparison of bacterial microbiota between CHD-NAFLD patients and HCs at species level. (**f**) The comparison of bacterial microbiota between CHD-NAFLD patients and CHD patients at species level. The *Student’s t* test and STAMP was used. 71 samples were used in each analysis

The difference in microbiota between the CHD patients and the HCs was analyzed using the software-STAMP [18]. Compared with the HCs, the abundance of *Collinsella* (*p* = 0.009), *Collinsella aerofaciens* (*p* = 0.008), *Bacteroides stercoris* (*p* = 0.042), *Ruminococcus albus* (*p* = 0.023) was significantly reduced in the CHD patients, the abundance of *Ruminococcus gnavus* (*p* = 0.050), *Bacteroides dorei* (*p* = 0.024) increased significantly (Fig. 4). The characteristic flora was analyzed using indicating species. We found that the indicating species in CHD patients was *Ruminococcus albus* (*p* = 0.015) (Fig. 3b). Among the three groups, the *Ruminococcus albus* had the lowest abundance in the CHD patients. However, there are currently few reports on *Ruminococcus albus*.

### The characteristic of bacterial microbiota of the CHD-NAFLD patients

The software-STAMP [18] was used to analyze the difference in microbiota between CHD-NAFLD patients and HCs (Fig. 4c/4d/4e). Compared with HCs, CHD-NAFLD patients had significantly lower abundance of *Parabacteroides* (*p* = 0.018) and *Parabacteroides merdae* (*p* = 0.018).

Compared with CHD patients, the abundance of *Ruminococcus gnavus* (*p* = 0.043) and *Bacteroides dorei* (*p* = 0.010) was significantly lower in CHD-NAFLD patients. Compared with HCs, the abundance of *Bacteroides dorei* was also reduced in CHD-NAFLD patients, though it was not statistically significant (the abundance of *Bacteroides dorei* in CHD-NAFLD and HCs:0.4443 and 1.3039).

After the comparison with CHD patients and HCs, we used the indicating species analysis to find the characteristic microbiota of CHD-NAFLD patients. The indicating species analysis found that the indicating species of CHD-NAFLD patients was *Bacteroides coprocola* (*p* = 0.016) and *Bacteroides fragilis* (*p* = 0.064) (Fig. 3b). Compared with the other two groups, the abundance of *Bacteroides coprocola* was the highest and the abundance of *Bacteroides fragilis* was the lowest in CHD-NAFLD patients.

### Correlation analysis between clinical indexes and bacterial microbiota at genus and species levels

**Fig. 5:**
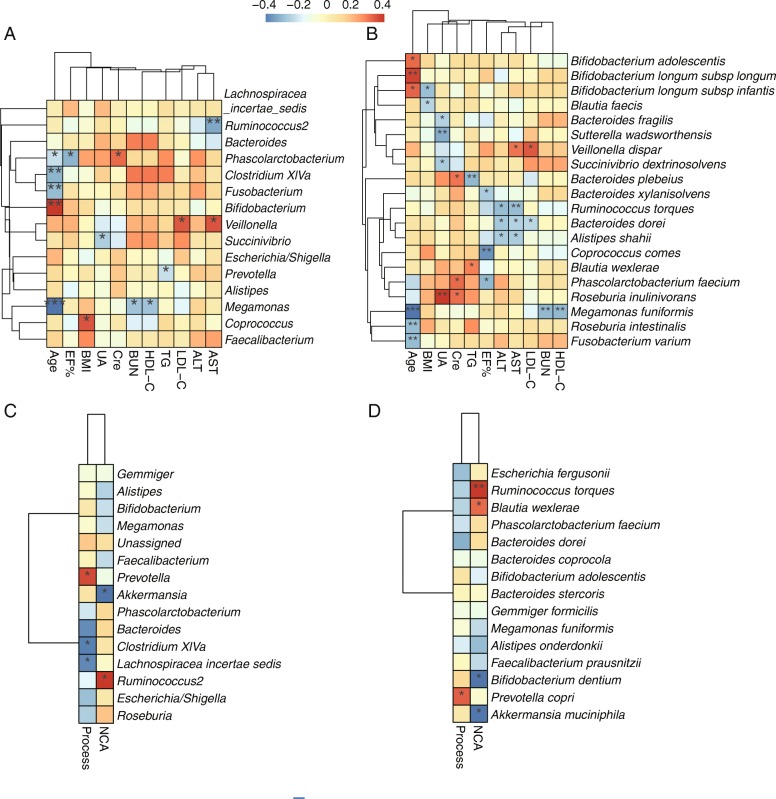
The correlation analysis between the clinical indexes and the microbiota. (**a**) The correlation analysis between clinical indexes and bacterial microbiota at the genus level. (**b**) The correlation analysis between clinical indexes and bacterial microbiota at the species level. (**c**) The correlation analysis between the severity of CHD and bacterial microbiota at the genus level. (**d**) The correlation analysis between the severity of CHD and bacterial microbiota at the species level. “Process” stands for the time from the initial diagnosis of CHD to this day. The Spearman’s correlation analysis was performed and R 3.5.1 software with *pheatmap* package was use for visualization. The scale bar in heatmaps stands for the correlation coefficient in the Spearman’s correlation analysis. *p < 0.05; **p < 0.01. NCA, Narrowed coronary artery. 71 samples were used in each analysis

The bacterial microbiota of all samples was included and Spearman’s correlation analysis was performed between bacterial abundance and clinical indexes (Fig. 5).

As mentioned above, there was no significant difference in BMI levels between patients with CHD patients and HCs and BMI levels were significantly higher in CHD-NAFLD patients. BMI positively correlated with the abundance of *Coprococcus* (*p* < 0.05) and negatively correlated with the abundance of *Bifidobacterium longum subsp infantis* (*p* < 0.05) (Fig. 5a and b). The abundance of *Coprococcus* was increased in CHD-NAFLD patients (the abundance of *Coprococcus* in CHD-NAFLD, CHD, HCs: 2.4892, 1.3141, 1.4745). The abundance of *Bifidobacterium longum subsp infantis* was lower in CHD patients than in HCs and was much lower in CHD-NAFLD patients (the abundance of *Bifidobacterium longum subsp infantis* in CHD-NAFLD, CHD, HCs: 0.0020, 0.04248, 0.1946).

Among the participants, UA increased in CHD-NAFLD patients. Correlation analysis showed that UA positively correlated with the abundance of *Roseburia inulinivorans* (*p* < 0.01) and negatively correlated with the abundance of *Sutterella wadsworthensis* (*p* < 0.01) and *Bacteroides fragilis* (*p* < 0.05) (Fig. 5b). Compared with CHD patients and HCs, the abundance of *Roseburia inulinivorans* was the highest in CHD-NAFLD patients (the abundance of *Roseburia* in CHD-NAFLD, CHD, HCs: 6.2419, 4.6199, 3.1823). The abundance of *Bacteroides fragilis* and *Sutterella wadsworthensis* was also the lowest in CHD-NAFLD patients (the abundance of *Bacteroides fragilis* in CHD-NAFLD, CHD, HCs: 0.13732, 0.7190, 0.4431; the abundance of *Sutterella wadsworthensis* in CHD-NAFLD, CHD, HCs: 0.1305, 0.1496, 0.2934).

The abundance of *Veillonella* positively correlated with AST (Fig. 5a). The abundance of *Veillonella* was higher in CHD patients than in HCs and was further increased in CHD-NAFLD patients (the abundance of *Veillonella* in CHD-NAFLD, CHD, HCs: 1.9087, 1.3366, 0.2289).

The ejection fraction of the overall CHD patients was lower than that of HCs. Correlation analysis found that the ejection fraction also negatively correlated with the abundance of *Phascolarctobacterium* (*p* < 0.05) and *Phascolarctobacterium faecium* (*p* < 0.05) (Fig. 5a and b). The abundance of *Phascolarctobacterium* in CHD-NAFLD patients and CHD was higher than that of HCs (the abundance of *Phascolarctobacterium* in CHD-NAFLD, CHD, HCs: 0.8941, 1.2038, 0.8541).

The abundance of *Bacteroides dorei* negatively correlated with ALT and AST (*p* < 0.05) (Fig. 5b). As mentioned above, the abundance of *Bacteroides dorei* was the lowest in CHD-NAFLD patients.

The results suggests that the abundance of some probiotics (such as *Bifidobacterium longum subsp infantis*) was reduced in CHD-NAFLD patients. On the other hand, the abundance of some bacteria (such as *Coprococcus*) that could promote metabolic syndrome increased in CHD-NAFLD patients. Correlation analysis indicated that the changes in abundance of these bacteria correlated with changes in clinical indexes such as BMI. It suggests that the abundance change of the bacteria mentioned above might be related to the changes of clinical indicators in CHD-NAFLD patients and could be one of the important factors affecting the degree of metabolic disorders. In the picrust analysis, the metabolism in the CHN-NAFLD patients and CHD patients was also different (Additional file 1).

### Correlation analysis between the severity of CHD and gut microbiota

The microbiota of all samples was included and Spearman’s correlation analysis was performed at the genus level and species level (Fig. 5c and d). At the genus level, the number of narrowed coronary artery negatively correlated with the abundance of *Akkermansia* (*p* < 0.05) and *Akkermansia muciniphila* (*p* < 0.05). Previous study reported that *Akkermansia* was protective for metabolism [19]. In this study, the abundance of *Akkermansia* in CHD-NAFLD patients and CHD patients was lower than that in HCs (the abundance of *Akkermansia* in CHD-NAFLD, CHD, HCs: 0.8778, 0.7938, 2.4593), which was consistent with the previous study [20].

## Discussion

The change of intestinal microbiota was an important factor in the occurrence and progression of CHD and NAFLD. At present, most of the research focused on the characteristics of the microbiota in CHD patients and NAFLD patients alone. There was no research on the characteristics of intestinal microbiota in CHD-NAFLD patients. Therefore, this study analyzed the characteristics of the microbiota of CHD-NAFLD patients from the perspective of bacterial microbiota.

In this study, there was no significant difference in the ɑ diversity of the microbiota between the overall CHD patients and HCs. The β diversity was somewhat different from HCs, but it was not statistically significant. At present, the diversity of the overall CHD patients and HCs was still controversial in previous studies, and the differences in diversity reported by different studies were not consistent [20, 21].

There was no significant difference in the α and β diversity of the CHD-NAFLD patients compared with CHD patients and HCs, which suggested that the richness and diversity of the microbiota had no significant difference between CHD patients, CHD-NAFLD patients and HCs. The diversity of gut microbiota represents the homeostasis of gut microbes and have been reported to be correlated with human health [22],but it was not positively correlated with the human health in all cases [23–25].

At the phylum level, the dominant bacteria on the bacterial microbiota of CHD-NAFLD patients and CHD patients were Firmicutes, Bacteroidetes and Proteobacteria. At the genus level, the abundances of *Bacteroides* and *Coprococcus* were relatively high in the overall CHD patients, which was consistent with the top 20 bacteria in abundance of the overall CHD patients reported in previous literature [20].

We firstly compared the overall CHD patients with HCs. In the overall CHD patients, we found that the abundance of *Collinsella* and *Parabacteroides* decreased compared with HCs, which was the characteristic for the overall CHD patients. It was reported that the abundance of *Collinsella* was enriched in people with metabolic diseases [26] and negatively correlated with dietary fiber intake in diet [27]. In addition, *Collinsella* was positively associated with hyperlipidemia in human, while the abundance of *Collinsella* was decreased significantly after atorvastatin treatment [28]. In this study, the all overall CHD patients was taking statin lipid-lowering therapy, while only 1/2 HCs took statin as lipid-lowering therapy. It was speculated that decreased abundance of *Collinsella* in the overall CHD patients might be related to the treatment of statins.

*Parabacteroides* was mostly reported as metabolic protective bacteria and have been shown to have protective effects on atherosclerosis and NAFLD in animal experiments [16]. The abundance of *Parabacteroides* was significantly reduced in mice with metabolic syndrome [16] and correlated with cardiac function and metabolism in mice [29]. It could be seen that the reduction of the abundance of protective bacteria *Parabacteroides* in the overall CHD patients was an important feature of the bacterial microbiota disorder, which was consistent with previous studies [20].

The intestinal bacterial microbiota in CHD-NAFLD patients showed an increase in the abundance of *Copococcus* and *Veillonella*, and a reduction in the abundance of *Bifidobacterium longum subsp infantis*, *Parabacteroides*, *Bacteroides fragilis*, *Ruminococcus gnavus* and *Bacteroides dorei*.

Among them, the abundance of *Ruminococcus gnavus* and *Bacteroides dorei* in CHD-NAFLD patients was significantly lower than that in CHD patients. At present, the functional reports on *Ruminococcus gnavus* are still controversial. It was positively correlated with dietary polyunsaturated triglycerides and obesity [30, 31]. On the other hand, the protective effect of the bacteria on health was also mentioned. The abundance of *Ruminococcus* was reduced in patients with cerebral infarction and cerebral ischemia. In obese mice, the increase in the abundance of *Ruminococcus* could promote the glucose metabolism and play an important role in sugar metabolism [32]. Therefore, in this study, *Ruminococcus gnavus* might show different characteristics in two different diseases of CHD-NAFLD and CHD and further research was needed. Correlation analysis found that the abundance of *Bacteroides dorei* negatively correlated with ALT and AST. Recent studies reported that *Bacteroides dorei* might inhibit atherosclerosis by reducing the production of intestinal microbial lipopolysaccharide [33]. A Japanese study reported that its abundance in CHD patients was reduced [34], but the sample size was relatively small (11 patients). In this study, the abundance of *Bacteroides dorei* in CHD patients was higher than that in HCs, which was different from the literature report and may be related to factors such as race, environment, diet and different inclusion criteria.

Correlation analysis found that uric acid levels negatively correlated with the abundance of *Bacteroides fragilis*. *Bacteroides fragilis* was mostly reported as the metabolically protective bacteria, and the abundance was significantly lower in NAFLD and metabolic syndrome populations than in healthy populations [35, 36]. In the study for metabolic diseases, an increase in the abundance of *Bacteroides fragilis* after vegetarian intervention was observed [37].

Correlation analysis suggested that BMI positively correlated with abundance of *Coprococcus* and negatively correlated with the abundance of *Bifidobacterium longum subsp infantis*. *Coprococcus* was reported to be related to metabolic syndrome, fatty liver and atherosclerosis. And it might be involved in the progression of metabolic syndrome [38]. In fatty liver mice, serum ALT positively correlated with the abundance of *Coprococcus* [39]. Moreover, *Coprococcus* was reported to positively correlate with the lipid levels and peroxidation of the macrophage in the apoE^−/−^ mouse model [15]. The abundance of *Bifidobacterium longum subsp infantis* was lower in CHD patients than in HCs and was further decreased in CHD-NAFLD patients. *Bifidobacterium longum subsp infantis*, reported as a probiotic, could significantly reduce liver fat accumulation, total cholesterol and lipid deposition and had a positive influence on the disease progression of NAFLD [40]. Oral administration of the bacteria could also significantly reduce serum total cholesterol and low-density lipoprotein cholesterol in children with dyslipidemia [14]. The abundance of *Bifidobacterium longum subsp infantis* significantly reduced in patients with metabolic syndrome [35]. It was observed as a probiotic to reduce post-myocardial depression and apoptosis in mice [41]. It suggests that the changes in the abundance of *Coprococcus* and *Bifidobacterium longum subsp infantis* in CHD-NAFLD patients might be an important factor of its metabolic disorder, which is related to the change of BMI. However, further research is needed.

The abundance of *Veillonella* positively correlated with AST. There were many reports that Veillonella was associated with atherosclerosis and metabolic syndrome, which was abundantly detected in arterial plaques [42]. It was reported that the abundance of *Veillonella* gradually decreased during the treatment and improvement of NAFLD [43]. It suggests that the increase in abundance of *Veillonella* in CHD-NAFLD patients might aggravate its metabolic disorder.

In terms of the severity of CHD, we selected the number of narrowed coronary artery in coronary angiography to assess the severity. Correlation analysis found it negatively correlated with the abundance of *Akkermansia*. At present, *Akkermansia* was considered a protective bacterium of atherosclerosis and was confirmed in mouse experiments [19]. Some Chinese herbal medicines could improve the abundance of *Akkermansia* and improve heart metabolic diseases [44]. A decrease in the abundance of *Akkermansia* was also observed in NASH patients [45]. In our study, the abundance of *Akkermansia* in the overall CHD patients was lower than that in HCs, which was consistent with previous studies [20, 21].

It could be seen that the reduction in abundance of metabolic protective bacteria such as *Parabacteroides*, *Bacteroides fragilis* and *Bacteroides dorei* in CHD-NAFLD patients may aggravate the degree of metabolic disorder.

Notably, this study is a correlation study and had no research on the function of the key differential bacteria. Thus, more studies are needed to confirm the function of the bacteria at the species level. Considering that our study was a single center study, more multi-center study is needed to the confirm the results.

## Conclusions

Changes of intestinal bacterial microbiota in CHD-NAFLD patients may be important factors affecting the degree of metabolic disorder, which may be one of the important reasons for the worse clinical outcome and disease progression in CHD-NAFLD patients than in CHD patients.

## Methods

### Subject enrollment

Patients who were admitted to the Department of Gastroenterology or Cardiology in Peking University People’s Hospital from January to September in 2018 were recruited. They must meet: (1) No viral hepatitis, autoimmune liver disease and alcoholic hepatitis. No chronic gastrointestinal disease and previous abdominal surgery; (2) Left ventricular ejection fraction ≥40% and no heart failure; (3) Age between 18 and 80 years. Women are not in pregnancy or abortion; (4) No antibiotics for nearly 2 weeks. No drinking alcohol, spicy food, yogurt and probiotics for nearly 1 week; (5) Normal stool frequency: 3 times / day - 3 times / week without diarrhea.

People were divided into three groups, including CHD patients (without NAFLD), CHD-NAFLD patients and healthy controls (HCs). The overall CHD patients included CHD patients and CHD-NAFLD patients. CHD diagnosis was confirmed by coronary angiography and individuals that had ≥50% stenosis in single or multiple vessels were included. NAFLD diagnosis was confirmed based on the evidence of hepatic steatosis via imaging [46]. B-ultrasound is the preferred method for imaging diagnosis of NAFLD [47]. Considering that liver biopsy was an invasive procedure, the guidelines recommended patients with undiagnosed NAFLD or suspected coexisting chronic liver disease needed the biopsy [47]. No such patients were included in this study. Therefore, this study mainly used B-ultrasound for imaging diagnosis of NAFLD. All the healthy controls enrolled were free of NAFLD, CHD and had none clinically CHD evidence such as angina and abnormal electrocardiographic.

The CHD-NAFLD patients were 1:1 matched with CHD patients and HCs according to the gender and age (±5). All the patients would receive abdominal ultrasound and biochemical tests in the Peking University People’s Hospital. The coronary angiography for the overall CHD patients was also performed in the Peking University People’s Hospital. Demographic data and clinical information were carefully collected.

### Sampling and sequencing

Fresh feces of each subject were collected after admission to the hospital. All samples were collected in Stool Collection Tube with Stool Satilizer and stored in − 80 °C freezers before further analysis in 48 h.

DNA was extracted from stool samples using the PSP® Spin Stool DNA Plus Kit protocol (Stratec, German). The full-length primer sequences, using standard IUPAC nucleotide nomenclature, to follow the protocol targeting this region are: 16S Amplicon PCR Forward Primer = 5′:TCGTCGGCAGCGTCAGATGTGTATAAGAGACAGCCTACGGGNGGCWGCAG, 16S Amplicon PCR Reverse Primer = 5’GTCTCGTGGGCTCGGAGATGTGTATAAGAGACAGGACTACHVGGGTATCTAATCC-3′ [48]. Each PCR product of the appropriate size was purified and quantified. And then, they were added to a master pool of DNA and analyzed using the MiSeq Reporter software and the MiSeq system.

### Sequencing data analysis

The main software used for sequence analysis is Vsearch v2.8.1 [49] and Usearch v10 (bit 32) [50]. The original data was merged using a double-ended sequence by Vsearch, followed by data quality control, excision of primers and barcodes. 14,108,373 sequences remained and 116,885 sequences were removed. Then we used Vsearch to remove the redundant sequences and sequences with < 30 occurrences. There are 6,372,406,955 base pairs in the 14,108,373 sequences with a minimum of 250 pairs and a maximum of 490 pairs (an average of 452 pairs). A total of 10,662,717 redundant sequences were removed and 17,741 high quality sequences remained.

The chimera was removed by ESV non-cluster de-noising [51] and Usearch v10 (balanced pattern) based on the reference sequence rdp_16s_v16_sp.fa and a total of 442 chimeric sequences were removed. 7505 non-chimera sequences were obtained. The Operational Taxonomic Unit (OTU) table was generated by Vsearch and the finally obtained sequence was clustered according to a certain threshold. The sequence of which the similarity is higher than 97% was defined as an OTU. In the 71 samples, a total of 7,570,391 reads (7434 OTUs) were obtained. Among these OTUs, 0 OTU appeared in all samples, 67 OTUs appeared in 90% of samples and 2931 OTUs appeared in 50% of samples. All samples were equally sampled to 30,000 reads with Usearch, resulting in a total of 2,124,467 reads (7434 OTUs). Among them, 0 OTU appeared in all samples, 42 OTUs appeared in 90% of samples and 1803 OTUs appeared in 50% of samples.

### Statistical analysis and visualization

The basic data were statistically analyzed using SPSS v21. Except for the special annotations, the measurement data were expressed as mean ± standard error (Mean ± SD). The data analysis between groups was analyzed by one-way ANOVA. A *p* value < 0.05 was considered statistically significant. The specific different statistical methods were described in the respective sections. Unless special annotations, the data was visualized by the ggplot2.

In the diversity analysis, Usearch was used for α and β diversity analysis [50]. Data differences were evaluated using the *Adnois* test.

In the difference analysis, we used the following methods: Using the STAMP software [52], the two groups of independent samples were compared using the *student’s t* test. A *p* value < 0.05 was considered statistically significant. A *p* value < 0.05 was considered statistically significant and the corresponding bacteria was included in the LEFSE analysis. Data visualization was achieved at the website (http://huttenhower.sph.harvard.edu [53]).

Random forest analysis was performed at the genus level using the *randomForest* package with a random seed of 315.

Indicator species analysis was performed on the genus and species levels using the *indicspecies* package, permutation = 999.

Spearman’s correlation was performed using the *psych* package and the *stringr* package, and the *p* value was corrected by the false discovery rate. Data visualization was performed using the *pheatmap* package. A *p* value < 0.05 was considered statistically significant and was labeled in the figure.

## Supplementary information

**Additional file 1.** The functional analysis in bacterial microbiota. Description: (A) The cellular processes in microbiota within the three groups. (B) The environmental information processing in microbiota within the three groups. (C) The genetic information processing in microbiota within the three groups. (D) The metabolism in microbiota within the three groups. (E) The organismal systems in microbiota within the three groups. (F) The human disease in microbiota within the three groups. The Phylogenetic Investigation of Communities by Reconstruction of Unobserved States (PICRUSt) and the greengenes database were used to predict the function of the microbiota. Kruskal−Wallis was used to compare the function prediction between groups.

## Data Availability

The datasets generated during the current study are available in the Sequence Read Archive (SRA), [https://www.ncbi.nlm.nih.gov/bioproject/PRJNA541489].

## Abbreviations

ALP: Alkaline phosphatase
ALT: Alanine aminotransferase
AST: Aspartate aminotransferase
BMI: Body mass index
BUN: Blood urea nitrogen
CHD: Coronary atherosclerotic heart disease
CHD-NAFLD: CHD patients complicated with NAFLD
GGT: Glutamyl transpeptidase
HCs: Healthy controls
HDL-C: High-density lipoprotein cholesterol
LDL-C: Low-density lipoprotein cholesterol
NAFLD: Nonalcoholic fatty liver disease
OTUs: Operational Taxonomic Units
PCoA: Principal coordinate analysis
PCR: Polymerase Chain Reaction
TG: Triglyceride
UA: Uric acid
