# Supplementary figures and images for: Changes of intestinal bacterial microbiota in coronary heart disease complicated with nonalcoholic fatty liver disease
Source: BMC Genomics. 2019 Nov 14;20:862. doi: 10.1186/s12864-019-6251-7 (PMC6857132; doi:10.1186/s12864-019-6251-7)

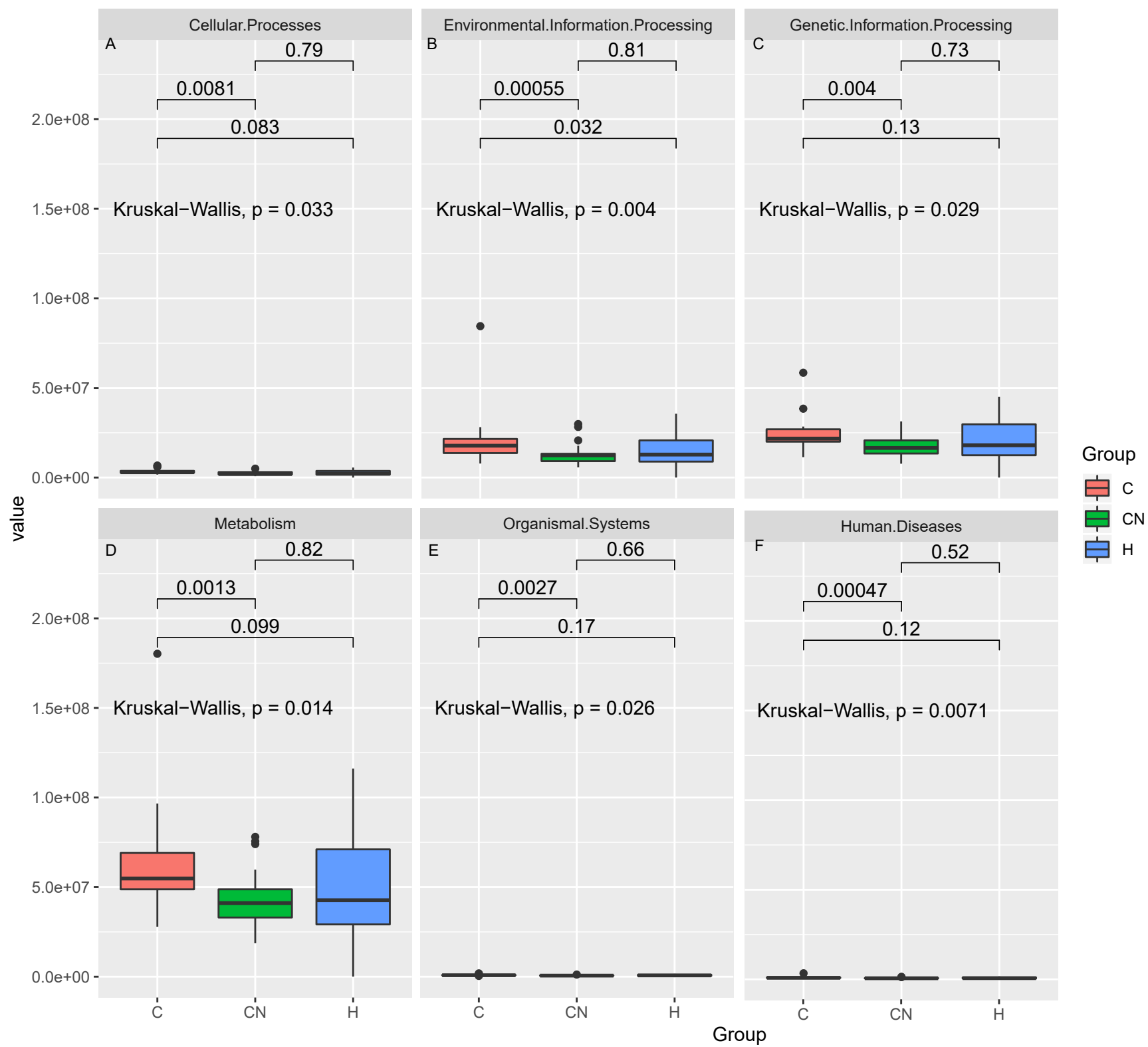

Supplement: Supplementary file 1 — Additional file 1. The functional analysis in bacterial microbiota. Description: (A) The cellular processes in microbiota within the three groups. (B) The environmental information processing in microbiota within the three groups. (C) The genetic information processing in microbiota within the three groups. (D) The metabolism in microbiota within the three groups. (E) The organismal systems in microbiota within the three groups. (F) The human disease in microbiota within the three groups. The Phylogenetic Investigation of Communities by Reconstruction of Unobserved States (PICRUSt) and the greengenes database were used to predict the function of the microbiota. Kruskal−Wallis was used to compare the function prediction between groups. [file 12864_2019_6251_MOESM1_ESM.pdf]
